# An Unusual Case of Hemosiderotic Fibrohistiocytic Lipomatous Lesion: Correlation of MRI and Pathologic Findings

**DOI:** 10.1155/2008/893918

**Published:** 2008-06-23

**Authors:** Ronald S. A. de Vreeze, Wim Koops, Rick L. Haas, Frits van Coevorden

**Affiliations:** Department of Radiology, Pathology, Radiation Oncology, and Surgical Oncology, Netherlands Cancer Institute/Antoni van Leeuwenhoek Hospital (NKI/AVL), 1066 CX Amsterdam, The Netherlands

## Abstract

The spectrum of lipomatous lesions ranges from benign to highly malignant disease. Differentiation between these lesions is important to indicate prognosis and choose the most appropriate treatment. Hemosiderotic fibrohistiocytic lipomatous lesion (HFLL) is a rare subtype of lipomatous tumor. The diagnosis is usually based on clinical, histological, and immunohistochemical information. Where magnetic resonance (MR) imaging is a suitable modality to assess fatty tumors, no data is reported on MR imaging of HFLL. Here, the MR characteristics are described in correlation with pathologic findings in a case of HFLL in the left thigh, an unusual location.

## 1. INTRODUCTION

The spectrum of lipomatous lesions ranges from benign to highly malignant disease. Differentiation between these lesions is important to indicate prognosis and choose the most appropriate treatment [1].

The hemosiderotic fibrohistiocytic lipomatous lesion (HFLL) is first described by Marshall-Taylor in 2000 [2]. The incidence of HFLL is estimated to be less than 0.2% of all benign lipomatous lesions [2].

There is an ongoing debate about the resemblance of early pleomorphic hyalinizing angiectatic tumor (PHAT) and HFLL; some consider HFLL a precursor lesion of PHAT, implicating HFLL to be a neoplastic lesion [3, 4], others consider HFLL an individual more reactive lesion [5, 6].

Based on the cases described so far, HFLL is most common in middle aged females, however there is a wide age spectrum. HFLL is typically located on the distal extremities, particularly on the dorsal side of the foot and may be associated with venous stasis and trauma [3]. The median size at clinical presentation is 50 mm and ranges between 1 and 170 mm [2, 5]. Surgical removal is mainstay treatment for this lesion.

Local recurrences appear in approximately 50% of cases and become apparent within one year [2, 5]. Distant metastases have not been reported. Characteristic histopathological features are the spindled cells morphology and the presence of variably prominent hemosiderin pigment. The most common immunoprofile is diffuse staining of the spindled cell with CD34.

Previous data suggest that the appearance of lipomatous tumors on magnetic resonance (MR) images is helpful in establishing a diagnosis [7, 8]. To our knowledge, there are no other reports describing the radiologic appearance of HFLL. We report on the imaging features in correlation with pathologic findings in a case of HFLL in the left thigh, an unusual location.

## 2. CASE REPORT

A 66-year-old Caucasian man was sent to our tertiary referral center for a lesion of the left thigh, nagging pain, uncertain radiological diagnosis without histologic diagnosis. The patient had noticed the lesion one and a half year before and it had slowly increased in size. Besides oral anticoagulation treatment for atrial fibrillation, there was no relevant medical history, specifically no trauma. Family history was noncontributory. Physical examination revealed a resistance involving half the anterior medial side of the thigh. MR imaging was performed.

**Figure 1:**
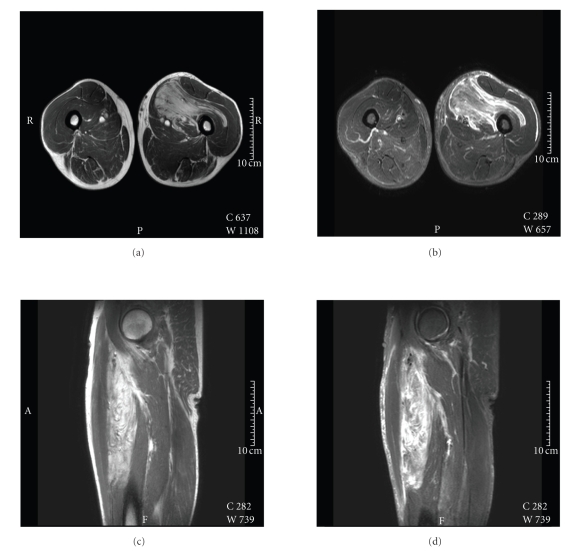
Upper left transversal T1 (TR 545 TE 20) and fat saturated STIR image (TR 12000 TI 200 TE60) upper right, showing the thigh and a homogenous lobulated mass. Coronal T1 and T2 weighted image is shown at the lower part of Figure 1, an image through left thigh showing a homogenous mass with irregular boundaries.

*MR imaging*, by Philips 3T Achieva and intravenous contrast series with Dotarem, showed a lipomatous lesion of the left thigh measuring 19 × 8 × 4 cm with irregular boundaries. The lesion showed multiple far reaching intramuscular and subfascial extensions. The assessment of internal structures showed a homogeneous, lobulated lesion. Figure 1 illustrates the high signal intensity of the lesion on T1- and T2 (STIR) weighted images with foci of hyperintensity on the fat-saturated (STIR) images. The signal intensity, particularly on T1 weighted images, was substantially lower than that of surrounding subcutaneous lipomatous tissue. Dynamic MR imaging was performed to characterize the enhancement pattern of the tumor, which showed homogeneous enhancement.

These combined imaging features were suggestive for a benign lesion or low grade sarcoma. However, we could not unequivocally define these MR images to a specific diagnosis. As intermediate or high grade sarcoma could not be ruled out, and these lesions in our institute are preferably treated by preoperative radiotherapy, a trucut biopsy was performed. A thoracic computed tomography scan was made which did not show distant metastasis.

Histopathological analysis did not allow a definitive diagnosis and suggested a not otherwise classifiable benign or low-grade lipomatous lesion; an intermediate or high-grade liposarcoma was unlikely.

Based on these findings, a surgical resection was planned.

The macroscopic aspect at surgery was a yellow-brown fatty gelatinous lesion, 19 cm in diameter, poorly circumscribed, unencapsulated and extending along muscles and neurovascular structures. A resection leaving no macroscopic residue (R1) was performed.

## 3. HISTOPATHOLOGICAL ANALYSIS

**Figure 2:**
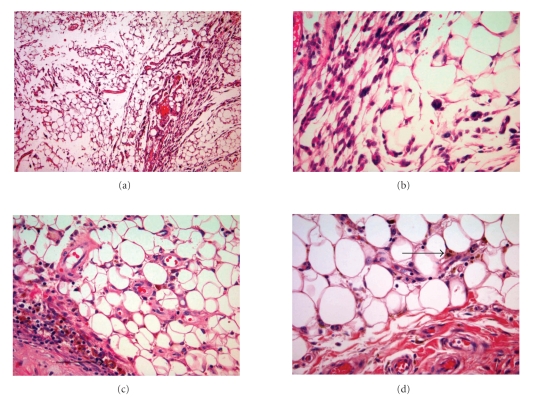
The upper left image shows the histology of HFLL. The upper right magnified image shows the spindled cell component of HFLL, whereas the inflammatory component of HFLL is shown in the lower left image, and macrophages with hemosiderin pigment (arrows) are shown in both the lower images. In all illustrations, mature adipocytes are present.

After resection specimens were histopathologically evaluated. Macroscopically, the unencapsulated lipomatous lesion showed tissue that was darker yellow than the normal surrounding fat. Microscopically, twenty representative samples throughout the whole tumor were reviewed on hematoxylin-eosin stained slides. In all of them, similar findings were observed: the lesion demonstrated mature adipose tissue with foci of proliferation of bland floret-, spindled-, and giant- cell areas, extending along and between muscle fibers. A lobular appearance was created by various small wide blood vessels associated with thin fibrous septa. Around these vessels, histiocytes, mast and plasma cells revealed the inflammatory parts of this tumor. The nuclei of the spindled and multinucleated cells varied in diameter, the latter having a Touton giant cell-like character. Occasionally larger nuclei were identified but without atypia, and very sporadically mitoses were seen. Granular brown to golden pigment and occasionally hyperchromatism was observed in the interstitial space of tumor (see Figure 2).

Immunohistochemical analysis showed a notable staining primarily of the spindled cells and multinucleated cells for CD34. S-100 was positive in mature fat cells. CD31, PPAR*γ*, and P53 were negative.

By combining histology and immunohistochemistry of resected specimen with clinical information, the diagnosis HFLL following Marshall-Taylor and Brown’s classification was established [2, 5].

## 4. DISCUSSION

The discrimination between the various differential diagnoses of fatty tumors is generally based on clinical, imaging, and histopathological features, sometimes combined with immunohistochemical or molecular genetic features. Besides clinical features suggesting malignancy, such as older age, large size, fast growth-rate, there are MR features that suggest malignancy. These include presence of nodular and/ or globular or nonadipose mass-like areas and a decreased percentage of fat composition. The percentage and analysis of these nonfatty regions in these lipomatous tumors help to distinguish between benign or malignant lesions. Furthermore, two prior studies in lipomatous tumors have emphasized that infiltrative margins may suggest the diagnosis of benign lipomatous lesion rather than that of liposarcomas [9, 10].

This case, unlike most described HFLL lesions, lacked a history of prior trauma, presented at an unusual location and was large. However, the macroscopic aspect, morphological and immunohistochemical features were consistent with HFLL. The specific histological HFLL features could be correlated to the MR characteristics. The fatty component of the tumor was macroscopically darker brown than normal fat tissue and correlated with lower signal intensity of the HFLL compared to the surrounding subcutaneous fat. The absence of a fibrous capsule could be recognized with MR findings as well as the wide intramuscular extension. The internal tumor structures seen on MR imaging were a reflection of the characteristic fibrous bands in the overall histological architecture. The spindled cell CD34 positive areas were corresponding to the enhanced parts of the tumor on the MR images. The abundant homogenous adipose areas were reflecting on MR imaging in the lobulated areas. The scarce specific iron deposition seen at histopathological review however was not recognized on MR images or dynamic gradient echo sequence, since the signal intensity on MR was not decreased by iron deposition of HFLL. Furthermore, the typical inflammatory parts of HFLL could not be correlated with characteristic MR features.

Recently, Folpe and Weiss have proposed that early pleomorphic hyalinizing angietic tumor has a presumed identical histology to HFLL [4]. Yet, the pathogenetic relationship between these two lesions remains to be determined [3, 5, 6]. It must, however, be emphasized that general awareness of HFLL allows physicians to reach a correct diagnosis. The presence of only 26 HFLLs that have been histologically described so far could be the main obstacle for physicians to be aware of such lesions.

## 5. CONCLUSION

This report shows the MR images of an unusual case of HFLL.
